# Investigating the Dynamic Relationship Between Anxiety and Spatial Memory Using Autonomous Ecological Momentary Assessment

**DOI:** 10.21203/rs.3.rs-9728773/v1

**Published:** 2026-06-03

**Authors:** Claire Han, Kun Zhao, Linda Wang, Hongkun Zhu, Yangjia Li, Luca Kolibius, Angela Velazquez, Sandy Song, Amy Cami, Jerina Carmona, Marla Hamberger, Randy Auerbach, Catherine Schevon, Joshua Jacobs, Brett Youngerman

**Affiliations:** Columbia University; Columbia University; Columbia University Irving Medical Center; University of Chicago; University of Chicago; University of Chicago; Columbia University Irving Medical Center; Columbia University Irving Medical Center; Columbia University Irving Medical Center; Columbia University Irving Medical Center; Columbia University Irving Medical Center; Columbia University; Columbia University Irving Medical Center; University of Chicago; Columbia University Irving Medical Center

**Keywords:** ecological momentary assessment, ambulatory cognitive assessment, digital phenotyping, spatial episodic memory, anxiety, epilepsy monitoring unit, within-person design

## Abstract

Anxiety has been extensively studied in relation to memory, yet its dynamic association with spatial episodic memory in naturalistic clinical settings remains largely unexplored. We developed an anxiety-spatial-memory EMA protocol (asm-EMA) and deployed it in 30 epilepsy patients undergoing inpatient EEG monitoring, delivering combined momentary anxiety ratings and a validated spatial memory task pseudo-randomly every 90–150 minutes across multiple days. Subject-level asm-EMA means and session-to-session variability both correlated significantly with standard neuropsychological assessments, supporting the clinical validity of our design. Elevated within-person STAI-6 was selectively associated with faster retrieval responses, yet spatial memory accuracy was independent of all three anxiety measures, suggesting a shift in response strategy rather than memory impairment. Within-day anxiety showed short-term carryover between consecutive sessions, with little persistence beyond the next session. The asm-EMA protocol provides a feasible, autonomous framework for capturing moment-to-moment anxiety-memory dynamics in naturalistic settings.

## Introduction

It is well established that anxiety profoundly influences cognitive function, most notably memory. Indeed, attentional control theory (Eysenck et al., 2007) posits that elevated anxiety competes for the limited attentional resources required for successful memory encoding and retrieval. Meta-analytic work demonstrates a reliable negative association between self-reported measures of anxiety and working memory capacity (Moran, 2016). Further, anxiety disorders are associated with episodic memory impairment (Airaksinen et al., 2005), and anxiety symptoms prospectively predict memory decline in cognitively healthy older adults (Fung et al., 2018). Spatial working memory appears particularly vulnerable to anxiety (Shackman et al., 2006; Vytal et al., 2013), consistent with the hippocampus serving a dual role in both spatial cognition and anxiety regulation (Bannerman et al., 2014). Yet despite this body of evidence, the anxiety-memory relationship is far from settled. The working memory impairment robustly observed under controlled laboratory conditions does not replicate when anxiety is measured naturalistically in community samples (Lukasik et al., 2019), suggesting that documented effects may be driven primarily by clinical-level or experimentally induced anxiety rather than the everyday fluctuations most people actually experience. The effect is also paradigm-specific, with anxious individuals showing a recall bias for threatening material but no reliable bias in implicit memory or recognition (Mitte, 2008). Procedural factors such as retention interval further modulate any effect that does emerge, such that higher anxious arousal impairs memory across a waking retention interval but not when retention spans nocturnal sleep (Niu et al., 2025). Together, these findings suggest that anxiety does not uniformly impair memory.

This heterogeneity may reflect a structural limitation in how the anxiety-memory relationship has been studied. The bulk of existing evidence derives from single-session, between-subjects designs in which anxiety is either experimentally induced or indexed once as a trait, and memory is probed under tightly controlled conditions far removed from the sustained, fluctuating affective contexts of daily life. Such designs cannot resolve how momentary anxiety co-varies with memory performance within individuals as it fluctuates across hours and days. Whether the between-person associations measured in the laboratory index the same processes as within-person fluctuations in naturalistic settings remains an open question, and the two levels of analysis are known to diverge substantially in magnitude, and at times in direction (Fisher et al., 2018; Hamaker, 2012).

Characterizing the within-person relationship between anxiety and memory requires not only repeated measurement but is best studied with a within-person structure, in which each subject serves as their own control and the effects of momentary anxiety are partitioned from stable individual differences such as trait anxiety or baseline cognitive ability (Bolger & Laurenceau, 2013). This matters because whether a person's memory is worse on their more anxious days is a fundamentally different question from whether more anxious people perform worse overall, and collapsing these levels of analysis yields misleading estimates of effect size and generalizability (Hamaker et al., 2015). Ecological momentary assessment (EMA) characterizes a subject’s state at a particular moment in time in a natural environment (Shiffman et al., 2008), and evidence from naturalistic studies confirms it detects effects that laboratory paradigms cannot. In one two-week smartphone EMA study, morning stress anticipation predicted working memory deficits later that day over and above stressful events that actually occurred, a finding that no single-session design could have produced (Hyun et al., 2019). EMA is therefore well suited to characterizing how anxiety and memory co-vary within individuals across time.

Notwithstanding, EMA has rarely been combined with performance-based cognitive assessment, and spatial episodic memory in particular has received limited attention. Most EMA cognitive work has examined working memory, visuospatial attention or processing speed in community samples (Singh et al., 2023; Sliwinski et al., 2018), domains well suited to the brief tasks required for smartphone delivery. While both verbal and spatial memory depend on hippocampal function, the animal stress literature has consistently and specifically implicated spatial memory in stress-induced impairment (Conrad et al., 1996). The clinical inpatient setting introduces additional logistical demands, though existing work suggests these are tractable. Mobile mood assessment has been validated against standard clinical measures in outpatient settings (Fortea et al., 2023; Nahum et al., 2017). Repeated psychological self-assessment is feasible in an epilepsy monitoring unit, albeit without an accompanying cognitive measure (Michaelis et al., 2018). Whether the anxiety-memory associations observed in the laboratory hold at the within-person level under naturalistic clinical conditions therefore remains an open question.

The present study addresses this gap by combining intensive within-person EMA with a performance-based spatial episodic memory task in patients undergoing multi-day inpatient Epilepsy Monitoring Unit (EMU) monitoring, a setting in which sustained, naturalistic clinical stress unfolds in real time. Here we report findings from an asm-EMA (anxiety-spatial-memory EMA) protocol in which 30 EMU inpatients completed repeated assessments across multiple days of hospitalization. Each assessment combined momentary anxiety ratings (VAS and STAI-6) with a validated spatial episodic memory task (Donoghue et al., 2023; Miller et al., 2018). As an observational study, the present work characterizes how anxiety and spatial memory co-vary under naturalistic clinical stress and establishes asm-EMA protocol as a valid longitudinal affective-cognitive assessment tool in this population. We address three questions: (1) whether the asm-EMA protocol captures clinically meaningful variation in anxiety and spatial memory as validated against standard neuropsychological assessments; (2) how within-person fluctuations in momentary anxiety co-vary with concurrent memory performance; and (3) how anxiety state and spatial memory performance measured by asm-EMA protocol fluctuate across time.

The asm-EMA protocol is a core component of the broader Context-Aware Multimodal Ecological Research and Assessment platform, which integrates asm-EMA behavioral data with synchronized continuous intracranial recordings, physiological monitoring, and passively acquired data streams including audio, video, and smartphone digital phenotyping (Youngerman et al., 2024). The behavioral signals from asm-EMA sessions anchor these multimodal data streams, enabling the platform to identify the neural and physiological correlates of anxiety-memory states in real time and ultimately to trigger closed-loop neuromodulation of anxiety at behaviorally informed moments. Establishing that asm-EMA protocol reliably captures meaningful within-person variation in anxiety and spatial memory is therefore a necessary first step toward that larger goal.

## Methods

Our study used anxiety-spatial-memory ecological momentary assessment (asm-EMA) protocol to test the relationship between momentary anxiety and spatial episodic memory in epilepsy patients undergoing EEG monitoring. Participants completed repeated asm-EMA sessions throughout their hospitalization, each comprising a brief anxiety assessment battery followed by a spatial memory task. Sessions were delivered automatically at pseudo-randomized intervals across the day, allowing us to capture naturalistic fluctuations in anxiety state and cognitive performance within and across individuals.

### 2.1 Recruitment and Enrollment

Potential subjects were identified from patients with known or suspected epilepsy scheduled for admission to the New York-Presbyterian/Columbia University Irving Medical Center EMU for non-invasive scalp video electroencephalography (EEG) or intracranial video stereoelectroencephalography (SEEG). Preliminary screening was conducted via electronic medical record review. Eligible subjects were aged 18–65, of any gender, proficient in English or Spanish, and had at least a 7th-grade reading level. Exclusion criteria included significant hearing, visual, or intellectual impairment; history of electroconvulsive therapy or psychosis (excluding postictal psychosis); lifetime history of psychotic disorder; current severe or unstable anxiety, major depressive disorder, or bipolar disorder; neurodegenerative disease or widespread brain lesions; language impairment; or medical conditions potentially affecting cognition (HIV, metastatic cancer, acute renal failure, or end-stage renal disease).

Once a subject was deemed eligible, the research team notified the treating physician to confirm appropriateness for enrollment. SEEG patients were contacted by phone prior to admission to introduce the study, while scalp EEG patients were approached on the day of admission in the EMU. Formal written consent was obtained in person, typically on the day of admission. The study was approved by the Columbia University Institutional Review Board (Protocol #: AAAU7374).

### 2.2 Anxiety-Spatial Memory Ecological Momentary Assessment

#### VAS Anxiety, VAS Pain & STAI-6.

**Figure 1:**
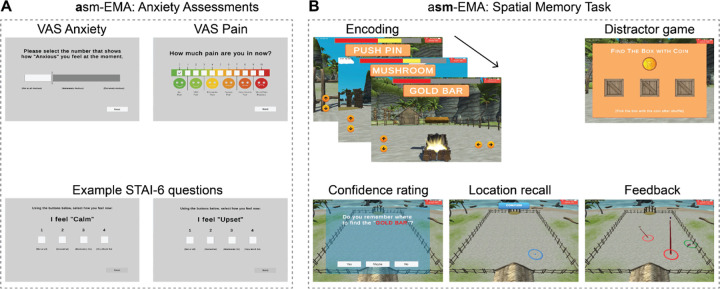
Structure of an anxiety-spatial-memory ecological momentary assessment (asm-EMA) session. Each asm-EMA session comprised two components delivered sequentially on a guided-access tablet. (A) Anxiety assessment. Participants completed a visual analog scale (VAS) for anxiety (top left), a VAS for pain (top right), and six items from the abbreviated State-Trait Anxiety Inventory (STAI-6; bottom), with example items shown for an anxiety-present (“I feel Upset”) and an anxiety-absent (“I feel Calm”) question. (B) Treasure Hunt spatial episodic memory task. In the encoding phase, participants navigated a virtual beach arena to locate treasure chests, each revealing an object (e.g., Push Pin, Mushroom, Gold Bar). An intervening distractor phase required tracking a coin shuffled between boxes. During the retrieval phase, participants indicated their confidence in remembering each object’s location before tapping the recalled position; corrective feedback displayed both the chosen (blue circle) and correct (red circle) locations.

During each asm-EMA session, patients completed three self-report instruments assessing current anxiety and pain. State anxiety was measured using a visual analog scale (VAS Anxiety), presented as a horizontal slider anchored at “Not at all anxious” (0) and “Extremely Anxious” (100). Current pain was measured with a similar VAS (VAS Pain), anchored at “No Pain” (0) and “Worst Pain Possible” (10), with emoticons accompanying the scale to aid interpretation. Both scales required participants to move a slider to indicate their current state (Fig. 1A top row).

Patients then completed the STAI-6 (Fig. 1A bottom row), a validated six-item abbreviation of the Spielberger State-Trait Anxiety Inventory designed for settings where the full 40-item form is impractical (Marteau & Bekker, 1992). The six items were selected from the 20-item state anxiety subscale based on their correlation with the remaining items, comprising the three highest-loading anxiety-present and three highest-loading anxiety-absent items. Each item is rated 1–4, with anxiety-absent items reverse-scored before summation, yielding a total score ranging from 6 to 24 where higher scores indicate greater state anxiety. The STAI-6 has been validated against the full STAI through concurrent administration across populations with both average and elevated anxiety levels.

#### Spatial Episodic Memory Task.

Following the anxiety assessment, participants completed the Treasure Hunt task, a spatial episodic memory paradigm involving virtual navigation, adapted from previous studies (Donoghue et al., 2023; Miller et al., 2018; Tsitsiklis et al., 2020). Using a touchscreen iPad, participants navigated a virtual beach arena (100 × 70 virtual units), encountered then later retrieved a series of treasure chests at randomized locations (Fig. 1B).

Each trial consisted of three phases. In the encoding phase, four chests appeared one at a time, and participants navigated to each in sequence. Upon reaching a chest, it opened to reveal either an object (displayed for 1.5s) or an empty interior; two or three of the four chests contained objects, and participants were instructed to remember their locations. A brief distractor phase followed, in which participants tracked a coin hidden under one of three boxes as the boxes repeatedly swapped positions. During the retrieval phase, participants were presented with each encountered object in random order and asked to rate their confidence in remembering its location (“Yes,” “Maybe,” or “No”) before tapping the remembered location directly on the arena. Responses within 11 virtual units of the true location were scored as correct, and point-based feedback was provided. Memory accuracy for each session was computed as the proportion of correct responses out of all objects attempted, where 0 is the worst possible response and 1 represents the best, following the scoring approach described in (Miller et al., 2018). Each session comprised 8 trials. Sessions with fewer than 4 completed trials were excluded, yielding 271 analyzable sessions across 30 participants.

### 2.3 Study Procedures

The asm-EMA session was delivered on a guided-access tablet (iPad Pro 11-inch, 2nd generation). Sessions were scheduled automatically by a custom server between 9:00 AM and 9:00 PM. Following study initiation, the first asm-EMA session was delivered after a random delay of 20–30 minutes, and subsequent asm-EMA sessions were delivered at randomized intervals of 90–150 minutes (ie. 2 hours +/− 30 minutes). Each session began with an audiovisual startle cue consisting of a 50-ms, 95-dB burst of white noise and a flashing tablet screen. Participants were required to answer the first three questions: current activity, VAS pain, and VAS anxiety, to silence the alarm, and could then choose whether to continue with the remaining STAI-6 items and the Treasure Hunt memory task. Responses were saved on a password-protected, HIPAA-compliant server accessible only to authorized study investigators.

### 2.4 Pre-admission and Post-discharge Questionnaires

At the beginning and end of the inpatient hospitalization period, subjects underwent a baseline evaluation of psychosocial wellbeing and environmental spatial ability by completing the Beck Anxiety Inventory (BAI), Beck Depression Inventory-II (BDI-II), Quality of Life in Epilepsy-31 (QOLIE-31), Spielberger State-Trait Anxiety Inventory (STAI), and Santa Barbara Sense of Direction scale (SBSOD). Details of each measure are provided in the supplemental material.

### 2.5 Data Quality and Exclusion Criteria

Sessions were excluded if the VAS pain or VAS anxiety ratings were not completed, or if fewer than 4 Treasure Hunt trials were completed. For the STAI-6, two additional response quality checks were applied. First, sessions were excluded if a participant gave identical responses to all six items, either all 1s or all 4s, as this pattern indicates uniform responding without regard to question content. Second, sessions were excluded if the time elapsed between consecutive STAI-6 item responses was less than 1 second throughout, suggesting the questions were not read. In total, 7 sessions were excluded based on these criteria, and the remaining 264 sessions were used in all analyses involving STAI-6.

### 2.6 Data analysis

First, we characterized the empirical distribution of each asm-EMA measure across completed sessions and quantified the relative contributions of between-subject and within-subject variation using intraclass correlation coefficients (ICCs) estimated from random-intercept linear mixed-effects models. This descriptive step was used to summarize the extent to which each asm-EMA measure reflected stable individual differences versus session-to-session fluctuation.

Second, to evaluate clinical validity, we computed Spearman correlations between subject-level asm-EMA summaries and pre- and post-monitoring neuropsychiatric trait assessments. Two asm-EMA summaries were considered for each measure: the subject-level mean, indexing average symptom level across the monitoring period, and the within-person standard deviation, indexing session-to-session variability. These analyses tested whether average asm-EMA responses and asm-EMA variability were associated with established clinical assessments collected before and after the study.

Third, to examine whether anxiety state and memory performance covaried within the same asm-EMA session, we analyzed both correlations and linear mixed-effects models among the asm-EMA measures. Between-subject correlations were computed on subject-level means, whereas within-subject correlations were computed on person-mean-centered session-level observations, so that stable between-subject differences were separated from momentary within-subject deviations. To formally test within-subject anxiety–memory relationships while accounting for repeated observations nested within participants, we fitted linear mixed-effects models with subject as a random intercept and person-mean-centered anxiety measures as fixed-effect predictors. Memory accuracy and response time were modeled separately, predictors were standardized to place coefficients on a common scale, and multivariable models were used to test whether any affective predictor explained unique variance beyond the others.

Last, to test whether asm-EMA measures showed short-term temporal structure, we examined within-day changes across repeated sessions. Cross-day session pairs and lag pairs separated by more than 5 hours were excluded from these analyses. Measures were person-mean centered and normalized by the pooled within-person standard deviation, so temporal effects reflected person-relative state deviations rather than between-subject differences in average level. We then computed within-person Spearman autocorrelations up to lag 3 to quantify same-measure persistence across sessions, and within-person Spearman cross-correlations to quantify lagged coupling between different asm-EMA measures. To test whether these lagged correlations reflected reliable temporal predictability rather than scheduling structure, we fitted mixed autoregressive models with subject-specific random intercepts and random slopes on the self lag-one term. These models tested whether each measure’s person-relative lag-one value predicted its current value after accounting for time of day and elapsed time between sessions. Mixed cross-regression models used the same framework to test whether lagged values of other asm-EMA measures improved prediction beyond each outcome’s own lag-one term. Within-subject permutation testing was used for all lagged analyses to determine whether observed effects depended on the true temporal ordering of sessions rather than shared marginal distributions.

## Results

### 3.1 asm-EMA Protocol Feasibility and Compliance

Over the course of hospitalization, 496 asm-EMA sessions were delivered across 30 inpatients (described in Table 1). Of these, 424 (85.5%) resulted in at least partial completion, 68 sessions (13.7% of all prompts) reached the VAS ratings only, and 85 (17.1%) reached the full anxiety assessment (VAS + STAI-6) but did not proceed to the spatial memory task. The remaining 271 sessions (54.6%) completed all three protocol components. 7 of these were subsequently excluded due to inattentive responding to the self-reported anxiety scores, yielding 264 sessions for data analysis. 28 participants contributed at least one complete session, and 23 completed three or more complete sessions which was the threshold we required for within-person modelling. Among participants with at least one complete session, the median number of asm-EMA sessions was 7.5 (mean = 8.80, range = 1–27).

**Figure 2:**
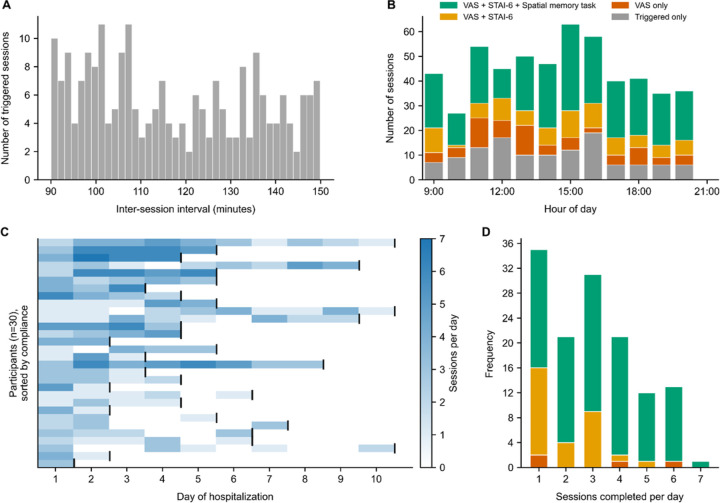
asm-EMA protocol compliance and session delivery characteristics. (A) Distribution of within-day inter-session intervals within the 90–150 minute triggering window. (B) Session completion by hour of day (09:00–21:00 EST), colored by completion tier: full protocol (VAS + STAI-6 + spatial memory task; green), full anxiety assessment (VAS + STAI-6; yellow), anxiety rating only (VAS only; orange), and triggered but not initiated (gray). (C) Session density per participant (rows, n = 30; sorted by total complete sessions) across relative hospitalization days (columns). White = no session; dark blue = highest daily session count. Vertical black lines indicate the end of the monitoring period. (D) Frequency of subject-days by number of sessions completed, colored by highest tier reached that day.

Compliance was sustained across the hospitalization rather than front-loaded (Fig. 2C). Participants who engaged consistently completed the full protocol on the majority of their active days, and the within-person drop-off typical of outpatient EMA studies was not evident here. Hospitalization length varied substantially across participants (range = 1–10 days), which accounts for much of the between-subject variance in total session count. The number of sessions completed per day ranged from 1 to 7 (Fig. 2D), with 1–4 sessions per day being the most common, consistent with the 90–150 minute inter-session triggering window.

Session completion was distributed across 9am–9pm (Fig. 2B). The full protocol constituted the largest category at nearly every hour of the day, indicating that time of day did not differentially drive dropout from the spatial memory component. Triggered-only sessions, in which prompts that were delivered but not initiated, were present throughout the day but did not cluster at any particular timepoint.

### 3.2 asm-EMA Protocol Validity and Variability

**Figure 3:**
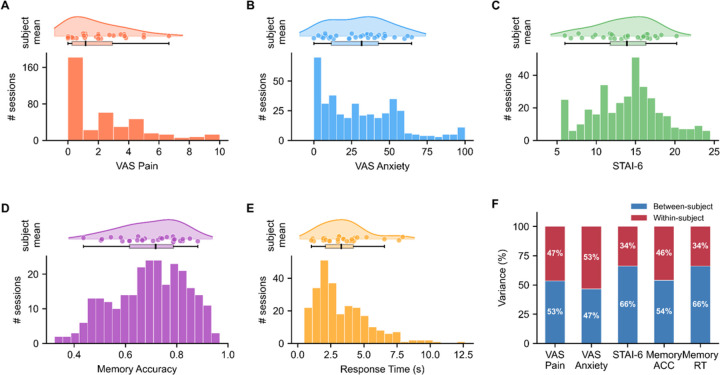
Session-level distributions and subject-level variability for all asm-EMA measures. (A-E): each shows two views of the same measure. The upper portion displays a raincloud plot of subject-level means, comprising a kernel density estimate, individual subject data points (jittered for visibility), and a box plot (median, IQR, and 1.5× IQR whiskers). The lower portion shows the session-level frequency distribution across all valid asm-EMA sessions. Measures shown are VAS Pain (A), VAS Anxiety (B), STAI-6 (C), Treasure Hunt memory accuracy (D), and Treasure Hunt response time (E). (F): shows variance decomposition for each measure, expressed as the percentage of total variance attributable to stable between-subject differences (blue) versus within-subject session-to-session fluctuation (red); the intraclass correlation coefficient (ICC), the proportion of variance between subjects, is numerically labeled within each bar segment where space permits.

Subject- and session-level distributions for all five asm-EMA measures are shown in Fig. 3. The two momentary anxiety measures showed markedly different distributions. VAS Anxiety was strongly right-skewed at the session level, with ratings concentrated near zero and a long tail extending to the scale maximum (Fig. 3B), consistent with an inpatient sample in which acute anxiety is episodic rather than sustained. STAI-6 scores were more uniformly distributed across the 6–24 range, with subject means spanning the full scale (Fig. 3C). This difference likely reflects measurement format where the single-item VAS is sensitive to acute distress but compresses responding at the low end, whereas the multi-item STAI-6 distributes variance more evenly by aggregating across items with differing affective content. VAS Pain showed the most pronounced floor effect of the three self-reported measures, with the majority of sessions rated at or near zero while a subset maintained persistently elevated pain levels throughout hospitalization (Fig. 3A). Memory accuracy was approximately normally distributed at the session level, centered near 0.75, indicating performance consistently well above chance (Fig. 3D). Response times were right-skewed, with a peak of 2–3 seconds and an extended upper tail, while subject means clustered between 2.5 and 5 seconds (Fig. 3E).

Variance decomposition via random-intercept models partitioned total variance into between- and within-person components. Between-person differences accounted for 53%, 47%, 66%, 54%, and 66% of total variance in VAS Pain, VAS Anxiety, STAI-6, memory accuracy, and response time, respectively (Fig. 3F). Notably, VAS Anxiety exhibited the largest within-person proportion (53%) despite its floor effect, likely because participants with near-zero baseline ratings produced disproportionately large session-to-session excursions on high-anxiety occasions. STAI-6 showed attenuated within-person variance (34%), consistent with item-level averaging dampening momentary reactivity relative to a single-item format. These divergent intraclass correlation profiles suggest the two instruments index partially distinct constructs where STAI-6 tracks stable individual differences across the trait spectrum, whereas VAS Anxiety is preferentially sensitive to acute state fluctuations. Across all five measures, within-person variance ranged from 34% to 53%, confirming sufficient session-level variability to support examination of within-person anxiety–memory covariation.

### 3.3 asm-EMA Session Mean and Variability Align with Standard Neuropsychological Assessments

**Figure 4:**
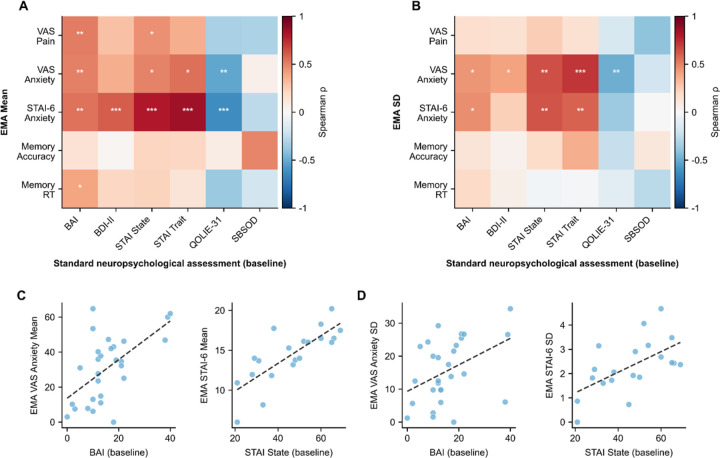
Convergent validity of asm-EMA means and within-person variability against standard neuropsychological assessments. Heatmaps show Spearman correlations between subject-level asm-EMA summary statistics (rows) and standard clinical measures administered at admission (columns): BAI, Beck Anxiety Inventory; BDI-II, Beck Depression Inventory; STAI State and Trait, State-Trait Anxiety Inventory; QOLIE, Quality of Life in Epilepsy; SBSOD, Santa Barbara Sense of Direction scale. Color encodes correlation magnitude and direction (red = positive, blue = negative); asterisks denote significance (* p < .05, ** p < .01, *** p < .001). (A) Correlations with asm-EMA subject means. (B) Correlations with asm-EMA within-person standard deviations. (C, D) Scatter plots illustrate representative associations between admission BAI and STAI State scores (x-axes) and asm-EMA VAS Anxiety mean and STAI-6 mean (C) and their within-person standard deviations (D). Dashed lines show ordinary least-squares fits.

Subject-level asm-EMA session means tracked standard neuropsychological assessments collected before and after the monitoring period (Fig. 4A; Fig. S2A; scatter plots of all significant correlation pairs in Fig. S3). STAI-6 showed the strongest and broadest correlation with the full STAI State and Trait inventories at ρ = 0.80–0.85 (Fig. 4C, right panel), with BAI (ρ = 0.54, p < .01) and BDI-II (ρ = 0.61, p < .001), and negatively with QOLIE-31 (ρ = −0.62, p < .001). The average VAS Anxiety and VAS Pain showed broadly similar positive correlations with BAI (ρ = 0.51, p < .006 for both) and STAI State (VAS Anxiety: ρ = 0.49, p < .027; VAS Pain: ρ = 0.46, p < .044). Among the memory measures, response time correlated with BAI (ρ = 0.40, p < .05), suggesting more anxious individuals taking longer to commit during retrieval. The trending positive correlation between spatial memory accuracy and the Santa Barbara Sense of Direction scale (SBSOD, ρ = 0.48, p < .078) suggests the task reflects genuine spatial cognitive ability.

Within-person variability (measured as standard deviation; SD) was also systematically predicted by baseline clinical severity (Fig. 4B). VAS Anxiety SD showed the broadest pattern of associations, correlating significantly with BAI (Fig. 4D, left panel), BDI-II, STAI State, STAI Trait, and QOLIE, indicating that patients with more severe baseline psychopathology fluctuated more from session to session. STAI-6 SD was more selective, associating significantly with STAI State (Fig. 4D, right panel) and STAI Trait but not with BDI-II or QOLIE, consistent with it indexing state anxiety reactivity more specifically than general affective severity. Neither memory measure nor VAS Pain showed significant SD associations with clinical instruments, suggesting that moment-to-moment fluctuations in pain and memory are driven more by proximal situational factors than by trait-level severity. The correspondence between asm-EMA measures and standard clinical instruments was maintained at follow-up, though the reduced sample at that timepoint limits the interpretive weight of those replications (Fig. S2; scatter plots of all significant correlation pairs in Fig. S4).

### 3.4 Covariation Between Momentary Anxiety State and Spatial Memory Performance

**Figure 5:**
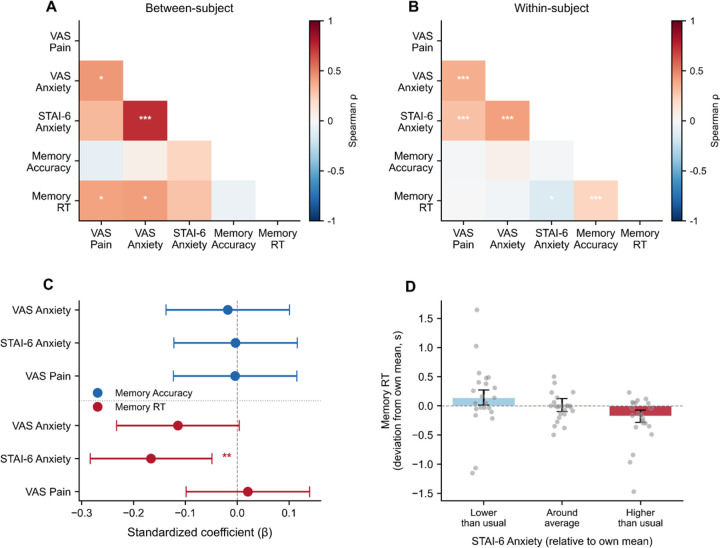
Within- and between-person associations among asm-EMA measures and their relationship to memory performance. Panels A and B show Spearman correlation matrices for between-subject and within-subject associations, respectively. (A) displays correlations computed on subject-level means; (B) displays correlations computed on person-mean centered session-level data. In both panels, only the lower triangle is shown and asterisks indicate significance (* p < .05, ** p < .01, *** p < .001); color encodes the Spearman ρ value. (C) shows a forest plot of standardized mixed-effects model coefficients (β ± 95% CI) for each affective predictor (VAS Anxiety, STAI-6, VAS Pain) on memory accuracy (blue) and response time (red), estimated from within-person centered predictors with subject as a random intercept. The dotted horizontal line separates accuracy from response time models. (D) shows mean memory response time (deviation from each participant’s own mean) across tertiles of within-person STAI-6 anxiety, where “Lower/Around/Higher than usual” reflects whether a given session fell below, near, or above that participant’s mean anxiety level. Bars show group means ± SEM; gray dots show individual subject means within each bin (one subject with response time deviation > 2 s excluded from display).

We examined anxiety-memory covariation at both the between-subject level (subject means) and within-subject level (person-mean centred values) using Spearman correlations (Fig. 5A–B, full pairwise scatter plots in Fig. S5), and tested within-person anxiety-memory associations using mixed-effects models (Fig. 5C). At the between-subject level, neither memory accuracy nor response time was significantly associated with any anxiety or pain measure, indicating that individuals who were generally more anxious or in more pain did not differ systematically in overall memory performance (Fig. 5A). The within-person pattern diverged sharply. On sessions when participants felt more anxious or in more pain than usual, momentary ratings co-elevated across all three affective measures (ρ = 0.27–0.33, all p < .001; Fig. 5B). This within-person affective coherence, in the absence of corresponding between-person correlation, suggests the three measures track a shared transient state rather than independent trait dimensions. Despite this coherence, within-person fluctuations in anxiety and pain showed no reliable association with concurrent memory accuracy for any predictor.

Mixed-effects models confirmed this dissociation (Fig. 5C). Memory accuracy was unrelated to any anxiety measure. Response time, however, was selectively associated with within-person STAI-6 fluctuations (β = −0.19, p < .01), an effect that survived covariate control for VAS Anxiety and VAS Pain (β = −0.17, p < .05). Counterintuitively, elevated anxiety was associated with faster, not slower, retrieval. Response times decreased monotonically across within-person STAI-6 tertiles by approximately 0.1–0.2 seconds (Fig. 5D), with no accompanying change in accuracy. This pattern is more consistent with reduced deliberation under anxious arousal than with any genuine facilitation of memory retrieval. Overall, spatial memory accuracy proved largely decoupled from momentary anxiety state within individuals, and the one significant effect, faster responding under elevated STAI-6, did not alter performance. These results thus point to preserved episodic memory function even during periods of heightened distress.

### 3.5 Short-Term Temporal Persistence and Predictability of Anxiety and Memory Performance from asm-EMA

**Figure 6:**
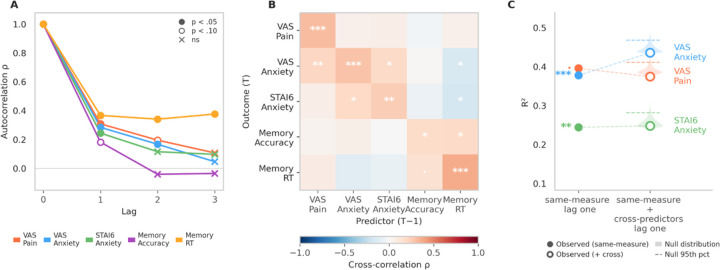
Temporal structure of asm-EMA measures. (A) Pooled within-subject Spearman autocorrelation (ρ) at lags 0–3 for all five measures. Filled circles indicate p < .05, open circles p < .10, and crosses non-significant, based on permutation testing (N = 2,000). (B) Lag-1 cross-correlation matrix (pooled Spearman ρ); rows = outcome at T, columns = predictor at T−1. Asterisks denote permutation significance (· p < .10, * p < .05, ** p < .01, *** p < .001). (C) Model comparison testing whether cross-predictors improved prediction beyond the same-measure lag-one model. Filled circles show R^2^ from the same-measure lag-one model; open circles show R^2^ after adding lag-one cross-predictors. Shaded areas show the within-subject permutation null distribution for the augmented model (N = 2,000), and dashed lines mark the null 95th percentile. Because the augmented model includes additional predictors, its permutation-null R^2^ is not expected to match the same-measure lag-one R^2^ exactly.

We next examined whether deviations from each participant’s usual asm-EMA level were carried forward from one session to the next. Under our sampling protocol of approximately 2-hour intervals, this allowed us to characterize short-term dynamics between consecutive assessments and evaluate whether the sampling frequency captured meaningful within-person change. We assessed these dynamics using within-person Spearman autocorrelations (more details in supplemental material), which tested whether person-relative values of the same measure were correlated across adjacent and later sessions (excluding cross-day transitions and unusually long gaps). Significance was evaluated using permutation testing (N = 2,000; Fig. 6A). Significant positive autocorrelation between adjacent sessions (lag one) was observed in four of five measures: VAS Pain (ρ = 0.31, p < .01), VAS Anxiety (ρ = 0.29, p < .01), STAI-6 Anxiety (ρ = 0.25, p < .01), and memory RT (ρ = 0.37, p < .001), with spatial memory accuracy showing a near-significant lag-one effect (ρ = 0.18, p < .10). Beyond lag one, autocorrelations were largely attenuated for the anxiety measures and spatial memory accuracy (Fig. 6A; Figure S6), indicating that temporal dependence was concentrated at the immediately preceding session. Response time was the exception, with significant persistence extending through lag three. Together, these lag profiles indicate statistically significant but modest short-term persistence in person-relative state deviations, with little evidence that this dependence extended to longer lags. Because these correlations were modest rather than large, repeated assessments appeared to capture related but not unchanged states.

Before interpreting these person-centered autocorrelations as evidence of state persistence, we tested whether within-person lag-one predictability remained after accounting for temporal features of the sampling schedule. This was motivated by the observation that anxiety measures varied by time of day (Figure S7), with morning sessions generally showing lower scores than evening sessions. We therefore fitted mixed-effects autoregressive models including time of day and elapsed time between sessions as covariates. Significance was evaluated against a within-subject permutation null that shuffled session order within each participant (N = 2,000; details in supplemental material). Three of five measures showed significant lag-one self-prediction beyond the null (Table 2): VAS Anxiety (R^2^ = 0.38, p < .001), STAI-6 Anxiety (R^2^ = 0.24, p < .01), and Memory RT (R^2^ = 0.37, p < .01). VAS Pain approached significance (R^2^ = 0.40, p < .10). No measure showed significant predictability from additional lags beyond lag one (p > .05), consistent with the lag-one-dominant structure observed in the autocorrelation analysis (Table ST1). Neither covariate showed significant effects on any measure (Figure S8). These results suggest that short-term predictability was not primarily explained by time of day or elapsed time between sessions, but reflected reliable within-person continuity in person-relative state deviations across adjacent assessments.

To test whether person-relative fluctuations in one domain were followed by changes in another, we examined cross-correlations among all five asm-EMA measures, asking whether person-relative deviations in one measure at a given session were correlated with person-relative deviations in another measure at a later session (permutation testing; details in supplemental material). Some anxiety measures showed significant cross-correlations with different measures at the following session (lag one); for example, higher-than-usual VAS Pain was correlated with higher-than-usual VAS Anxiety at the following session (ρ = .21, p < .01; Fig. 6B). Cross-correlations at longer lags are shown in Figure S9. These findings suggest possible short-term coupling between measures. However, cross-correlation alone cannot tell whether one measure contributes predictive information about later person-relative deviations in another, because an apparent lagged correlation may arise when each measure is persistent over time and the two measures are also correlated within the same session. We therefore extended the autoregressive models by adding lagged predictors from the other affective measures, referred to here as cross-predictors. For each anxiety outcome, we tested whether these cross-predictors improved prediction beyond the same-measure lag-one predictor. No significant cross-predictor effects were found for any measures (Fig. 6C). Adding the other affective measures from the previous session did not significantly improve model performance beyond the same-measure lag-one predictor (ΔR^2^ ≤ .01 for all outcomes; within-subject permutation testing, details in supplemental material). These results indicate that cross-correlations between different measures did not add predictive information once each outcome’s own previous-session person-relative value was accounted for. Thus, the main temporal pattern in the asm-EMA data was persistence of values within the same measure across adjacent sessions.

## Discussion

A central motivation of this study was to close the ecological validity gap in anxiety–memory research by measuring mood and memory together, repeatedly, and in context. The asm-EMA protocol is well positioned to this goal because it captures subject state in near-real-time in natural environments, reducing dependence on retrospective report and improving ecological validity (Shiffman et al., 2008; Stone et al., 2023). Prior naturalistic cognitive studies have established that brief smartphone-based tasks can be used outside the laboratory to obtain reliable cognitive measurements and to track meaningful within-person cognitive variation over time (Moore et al., 2016; Singh et al., 2023). The present findings extend that evidence to a clinical inpatient setting. The asm-EMA protocol produced interpretable, moment-to-moment measures of anxiety and spatial memory across multiple days of hospitalization, identifying substantial within-person variability in both domains.

Subject-level asm-EMA means showed strong correspondence with standard clinical assessments collected before (as baseline) and after (follow-up) the monitoring period. The STAI-6 correlated strongly with the full STAI State and Trait inventories, sufficient to justify its use as an efficient substitute for the full inventory in repeated inpatient monitoring. Spatial memory accuracy correlated positively with the SBSOD scale, confirming that the task indexes a distinct spatial cognitive ability rather than a proxy for affective state. Beyond mean level, asm-EMA protocol captured a second clinically meaningful dimension which is the within-person variability. Patients with higher baseline clinical anxiety showed greater moment-to-moment STAI-6 fluctuation during hospitalization, indicating that trait severity predicts not just where someone resides on the anxiety scale on average, but how much they fluctuate over time. A single clinical snapshot misses this dimension entirely. The correspondence between asm-EMA means and standard neuropsychological instruments was largely maintained at follow-up, though the reduced sample at that timepoint limits the interpretive weight of these replications.

Importantly, there was no evidence of response bias on the basis of anxiety level at the time of the EMA alert. If patients with greater anxiety were less likely to complete the full asm-EMA session, the anxiety scores in subjects completing the more extensive memory assessment would be systematically lower, biasing any anxiety-memory comparison. The data do not support this systematic dropout bias. Brief VAS Anxiety and VAS Pain assessments, mandatory to silence the alert, were completed after 85.5% of alerts, did not differ based on the extent of asm-EMA session completion (VAS Anxiety: Kruskal-Wallis H = 1.49, p > .474; VAS Pain: H = 2.02, p > .364; Fig. S1), with group means within 3 points on a 100-point scale. Within-session dropout was effectively independent of anxiety state at onset.

Momentary anxiety showed no significant association with concurrent spatial memory accuracy across all within-person specifications, including bivariate correlations and multivariate models adjusting simultaneously for all three affective measures. The between-person pattern was consistent, with patients who reported higher average anxiety or pain across their stay performed no differently on the spatial memory task overall. These findings extend prior laboratory work reporting anxiety-related memory impairment under controlled stress conditions (Shackman et al., 2006; Vytal et al., 2013) to a naturalistic clinical context, where the relationship may operate differently. Whether this reflects genuine memory resilience under ecological conditions, adaptation to chronic stress, or anxiety levels below the threshold for memory disruption remains an open question worth pursuing in future work.

Within-person STAI-6 predicted faster retrieval responses, and this association remained significant when VAS Anxiety and VAS Pain were included in the same model, confirming it was not driven by general distress. Response times decreased steadily across low, average, and high anxiety sessions with no sign of a threshold or reversal. Higher-than-usual anxiety shortened response time without affecting accuracy. One account consistent with attentional control theory (Eysenck et al., 2007) is that cognitive-evaluative anxiety prompts earlier disengagement from deliberative retrieval. Patients may commit to a location sooner when anxious, not because they retrieve more effectively but because they search less. The processing efficiency framework offers a compatible reading, distinguishing performance effectiveness from the cognitive effort required to achieve it and allowing for anxiety to compress deliberation time without altering retrieval outcome (Eysenck & Calvo, 1992). That the effect was specific to STAI-6 and absent for VAS Anxiety points to the cognitive appraisal component of anxiety as the operative mechanism rather than arousal or somatic distress.

Our temporal analyses suggest that asm-EMA protocol captured structured within-subject variation that was primarily local in time. In the emotion-dynamics literature, lag-one autoregressive carryover is often interpreted as emotional inertia, or the extent to which an affective state persists into the next measurement rather than changing immediately (Kuppens et al., 2010). More recent EMA-based emotion-dynamics work has extended this idea using autoregressive mixed-effects models to estimate how the strength of affective persistence and coupling may vary across individuals or over time (Pooseh et al., 2024). Within this context, our findings support a pattern of short-lived inertia rather than prolonged carryover: affective and behavioral states were related across adjacent sessions, but this dependence largely decayed beyond lag one. Thus, repeated asm-EMA measurements were not random from session to session; instead, the protocol appeared to capture a short-lived form of inertia that was strongest between immediately adjacent assessments.

A practical question raised by these findings is whether the roughly two-hour sampling interval was appropriate for capturing within-person state variation. In the present study, this interval was sufficient to detect lag-one persistence, suggesting that asm-EMA protocol captured meaningful within-person states over this time scale, such as whether anxiety, pain, or task reaction time remained higher than baseline roughly two hours later. Importantly, however, the optimal sampling frequency depends on the scientific question. Less frequent sampling may be sufficient for questions about broad daily state, such as whether a participant is generally having a better or worse day, whereas denser sampling may be needed to distinguish more fine-grained changes, such as a mild increase versus a sharp spike in pain or anxiety after a seizure, medication change, procedure, or other clinical event. A strength of the asm-EMA framework is that its automated scheduling system can be adapted to these different temporal scales, allowing future studies to tune the sampling interval to the dynamics of the target state.

To our knowledge, this is the first study to combine repeated ecological momentary anxiety assessment with a performance-based spatial memory task in a clinical inpatient population, and the first to characterize within-person anxiety-memory covariation under naturalistic clinical stress conditions. Prior EMA work in cognitive domains has relied almost exclusively on community samples and brief attentional tasks (Fortea et al., 2023; Nahum et al., 2017; Singh et al., 2023; Sliwinski et al., 2018). Extending this methodology to an inpatient epilepsy setting with a validated episodic memory paradigm represents a substantive step toward ecologically grounded cognitive monitoring in clinical practice.

Our findings carry several practical implications for inpatient EMU assessment. Response speed appears to be a more sensitive behavioral marker of acute anxiety than accuracy in this population. Patients experiencing elevated anxiety responded faster without any corresponding change in memory performance, which means clinicians interpreting bedside assessments during high-anxiety periods should not equate faster responding with better or worse memory function. The stability of accuracy across affective fluctuations is also reassuring for pre-surgical neuropsychological evaluations conducted during EMU admission. Within the range of anxiety levels observed here, elevated anxiety did not systematically depress memory scores. The strong correlation between asm-EMA ratings and full clinical inventories further suggests that repeated short assessments could realistically substitute for lengthy batteries in monitoring anxiety burden across a hospitalization, with the added advantage of capturing affective reactivity rather than just average severity.

Several limitations warrant consideration. The cohort was modest in size and protocol completion was uneven across participants, limiting statistical power for weaker effects and confidence in subgroup-level inference. The anxiety-memory findings are also conditional on task completion. Because participants could stop after the self-report portion, cognitive analyses may under-sample sessions marked by high distress or disengagement. VAS anxiety and pain scores did not differ significantly across completion tiers, providing some reassurance against systematic bias, though subtler forms of selective missingness cannot be ruled out completely. The present analysis also focused on average within-person temporal patterns rather than individual differences in affective dynamics. Prior work suggests that affective inertia varies substantially across individuals and is elevated in those with depressive symptoms (Kuppens et al., 2010), and characterizing this heterogeneity in future studies would enrich the picture considerably.

The clinical context in which our study was conducted introduces additional confounds that cannot be fully resolved here. Patients with epilepsy show elevated rates of anxiety and memory impairment relative to the general population (Scott et al., 2017), and those with temporal lobe involvement may show altered anxiety-memory dynamics specifically (Brown et al., 2023). Antiseizure medication withdrawal, standard practice during EMU monitoring, may independently modulate both anxiety and cognition across the monitoring period. Seizure timing relative to individual sessions was not systematically tracked, and post-ictal confounding of affective and cognitive ratings in a subset of observations cannot be excluded. Prospective studies should define and separately analyse sessions falling within a post-ictal window, as these states may differ substantially from interictal baseline.

Our study represents a foundational step within the broader Context-Aware Multimodal Ecological Research and Assessment platform (Youngerman et al., 2024), which aims to build a context-aware framework for tracking internal state using synchronized multimodal data including intracranial recordings, physiological monitoring, and passively acquired behavioral signals. Within that framework, the asm-EMA protocol serves two functions, as a standalone clinical assessment tool and as a source of structured behavioral ground truth for predictive modeling. The short-term temporal dependence observed between adjacent sessions is particularly relevant for the latter, since asm-EMA labels preserve local continuity rather than acting as isolated snapshots and provide a natural structure for training state-prediction models that learn from shared short-term context without redundancy. As the platform scales to larger samples and richer data modalities, the behavioral and clinical validity established here provides the empirical foundation on which multimodal predictive models can be built and interpreted.

## Supplementary Files

This is a list of supplementary files associated with this preprint. Click to download.

- EMAsupplement.docx

## Figures and Tables

**Table 1.** Demographic information and clinical characteristics for enrolled subjects. Of 33 enrolled subjects, 30 went on to participate in the study. Duration of participation refers to the number of days that at least one asm-EMA session was triggered. Seizure type is calculated as the percentage of total patients who seized during hospitalization who experienced the listed type of seizure. These categories are not mutually exclusive; most patients experienced more than one type of seizure.

	N	Percentage	Median	IQR	Range
Age	33		34	19	46
Sex					
Female	22	66.7			
Male	10	30.3			
Nonbinary	1	3			
Duration of participation (days)	30		4	2.8	9
Confirmed epileptogenic seizures					
Yes	21	70			
No	9	30			
Seizure localization					
Temporal	14	66.7			
Extratemporal	4	19			
Mixed	3	14.3			
Seizure type (% of total subjects who seized)*					
Spontaneous	21	100			
Focal impaired awareness	13	61.9			
Focal to bilateral tonic-clonic	10	47.6			
Subclinical	8	38.1			
Focal aware	3	14.3			
Generalized (absence)	2	9.5			
Stimulation-provoked	5	23.8			
Focal impaired awareness	3	14.3			
Focal aware	2	9.5			
Subclinical	1	4.8			
Baseline neuropsychological/spatial navigation assessment scores					
QOLIE-31	29		48	38	72
BDI-II	30		17	20	38
BAI	30		13	10.5	41
STAI state	22		47.5	28.5	55
STAI trait	22		42.5	21.5	42
SBSOD	16		4.15	1.75	5.2
Follow-up neuropsychological/spatial navigation assessment scores					
QOLIE-31	12		54	30.8	63
BDI-II	12		12.5	13.5	34
BAI	12		13.5	7	28
STAI state	12		47	16.5	49
STAI trait	12		42	18.5	33
SBSOD	12		3.9	1.5	4.8
EEG type					
Scalp	20	66.7			
Intracranial	10	33.3			

* Of note, all 21 patients who seized experienced a spontaneous seizure, and 5 of the 21 patients who seized also experienced at least one stimulation-provoked seizure.

**Table 2:** Autoregressive self-prediction at lag-1. R^2^ is the proportion of total within-person variance explained by the full mixed-effects model (M2: lag-1 score, elapsed time, time of day; random intercept and random lag-1 slope per participant). The null 95th percentile is derived from N = 2,000 within-person temporal permutations; p is the one-tailed permutation p-value (proportion of null R^2^ ≥ observed R^2^). Bold highlights statistical significance.

asm-EMA measures	Observed R^2^	Null 95 pct R^2^	P (shuffle)
VAS Pain	0.396	0.397	0.051
VAS Anxiety	0.378	0.275	**<0.001**
STAI-6 Anxiety	0.244	0.191	**0.003**
Memory Accuracy	0.136	0.260	0.567
Memory RT	0.372	0.294	**0.002**

## Data Availability

Data supporting the study's conclusions from the corresponding author is available upon reasonable request.

## Code availability

The analysis code used in this study is available from the corresponding author upon reasonable request.

## Abbreviations

EMA: ecological momentary assessment
asm-EMA: anxiety-spatial-memory EMA
VAS: Visual Analog Scale
STAI: Spielberger State-Trait Anxiety Inventory
STAI-6: six-item STAI
BAI: Beck Anxiety Inventory
BDI-II: Beck Depression Inventory-II
QOLIE-31: Quality of Life in Epilepsy-31
SBSOD: Santa Barbara Sense of Direction scale
EMU: Epilepsy Monitoring Unit
EEG: electroencephalography
SEEG: stereoelectroencephalography
ICC: intraclass correlation coefficient
RT: response time
